# A benign helminth alters the host immune system and the gut microbiota in a rat model system

**DOI:** 10.1371/journal.pone.0182205

**Published:** 2017-08-03

**Authors:** Laura Wegener Parfrey, Milan Jirků, Radek Šíma, Marie Jalovecká, Bohumil Sak, Karina Grigore, Kateřina Jirků Pomajbíková

**Affiliations:** 1 Departments of Botany and Zoology, University of British Columbia, Vancouver, Canada; 2 Integrated Microbial Biodiversity Program, Canadian Institute for Advanced Research, Toronto, Canada; 3 Institute of Parasitology, Biology Centre of the Czech Academy of Sciences, České Budějovice, Czech Republic; Universidade de Aveiro, PORTUGAL

## Abstract

Helminths and bacteria are major players in the mammalian gut ecosystem and each influences the host immune system and health. Declines in helminth prevalence and bacterial diversity appear to play a role in the dramatic rise of immune mediated inflammatory diseases (IMIDs) in western populations. Helminths are potent modulators of immune system and their reintroduction is a promising therapeutic avenue for IMIDs. However, the introduction of helminths represents a disturbance for the host and it is important to understand the impact of helminth reintroduction on the host, including the immune system and gut microbiome. We tested the impact of a benign tapeworm, *Hymenolepis diminuta*, in a rat model system. We find that *H*. *diminuta* infection results in increased interleukin 10 gene expression in the beginning of the prepatent period, consistent with induction of a type 2 immune response. We also find induction of humoral immunity during the patent period, shown here by increased IgA in feces. Further, we see an immuno-modulatory effect in the small intestine and spleen in patent period, as measured by reductions in tissue immune cells. We observed shifts in microbiota community composition during the patent period (beta-diversity) in response to *H*. *diminuta* infection. However, these compositional changes appear to be minor; they occur within families and genera common to both treatment groups. There was no change in alpha diversity. *Hymenolepis diminuta* is a promising model for helminth therapy because it establishes long-term, stable colonization in rats and modulates the immune system without causing bacterial dysbiosis. These results suggest that the goal of engineering a therapeutic helminth that can safely manipulate the mammalian immune system without disrupting the rest of the gut ecosystem is in reach.

## Introduction

Helminths are masterful immune regulators [1–3]. The absence of helminths is suggested to be linked to the global rise in immune-mediated inflammatory diseases (IMIDs) [4] and their restoration to the mammalian/human gut ecosystem shows promise in the prevention and, in some cases, treatment of IMIDs [5, 6]. IMIDs, such as Crohn’s disease, ulcerative colitis, type 1 diabetes or rheumatoid arthritis, now affect 7–9% of Westernized populations [7–9], and prevalence is increasing across the globe as the populations adopt Western lifestyles [10]. The rapid rise in IMIDs suggests that changes in environmental factors that impact the host immune system are at least partly responsible, and many lines of evidence implicate changes in the microbial environment [10, 11].

Helminths and the bacterial microbiota both influence the development of IMIDs [3] [12, 13]. Helminth infections are protective against IMIDs in many model systems (reviewed in [3, 14]) and treat disease symptoms in some [15, 16], though they are can cause harm in other ways [3, 17]. Understanding the impact of helminths on the rest of the gut ecosystem this is a rapidly expanding field [18]. This is an important consideration because gut bacteria directly influence IMIDs, which are characterized by dysbiosis and lower bacterial diversity [12, 13, 19], and reintroducing bacterial diversity (i.e., fecal transplantation) is sometimes an effective treatment for IMIDs [20]. These data give rise to the hypothesis that the therapeutic effect of helminth introduction partially results from shifts in the gut microbiota induced by helminth infection, which is supported by recent studies [21, 22].

Previous studies investigating the changes in the microbiota induced by helminth infection have reached divergent conclusions [18]. Some animal studies report major turnover in microbial community composition and decreases in alpha diversity (dysbiosis), particularly in mice infected with parasitic nematodes *Trichuris muris* or *Heligmosomoides polygyrus* that establish chronic infections [23–25]. In contrast, Broadhurst et al. [26] showed that *Trichuris trichiura* (whipworm) infection in macaques with gastrointestinal disease shifted the microbiota composition toward that observed in healthy animals and increased microbial diversity. Other studies in humans reveal small changes or no changes in the microbiota associated with the presence of varied helminths [27–29]. A previous study of *Hymenolepis diminuta* infection in rats found no change in alpha diversity, but a large compositional shift [30]. It is not clear what underlies the diversity of microbiota response to helminth infections. It may reflect differences in the type of infection (acute pathogen versus chronic infection versus benign long term colonization), the biology of the host response (e.g., mice may respond more forcefully than humans to helminths), the microbial environment of the host (laboratory environments with restricted microbial diversity versus the wild), or a combination of these and other factors. Identifying helminths that cause minimal disturbance to the microbiota, along with the environmental and host conditions that promote minimal disturbance are likely important to the success of helminth therapy.

Amidst the growing body of research on the human gut microbiome and helminth therapy it is important to build an integrated understanding of the impact of helminths on the gut ecosystem that captures the interactions between gut microbiome, benign helminths and the host immune system. Much research on helminth therapy investigates their interactions with the immune system separately from the changes in the gut microbiome after infection induction, though this is rapidly changing (e.g., [18, 21, 22]). Further, capitalizing on the promise of helminths as therapeutic agents for various IMIDs requires investigating of additional candidates [31]. Here, we assess the changes in the host immune system and changes in the gut microbiota in response to a benign helminth infection in a healthy rat model system.

We use the tapeworm *H*. *diminuta*, which establishes long-term asymptomatic infections in rats without altering the health of the rat [32], and has been shown to be protective against IMIDs in some laboratory models [33]. The immune reponse to *H*. *diminuta* infection in rats involves an initial induction of a type 2 immune response combined with with immunoregulation [33, 34], similar the immune response to helminths generally [35]. By using a benign helminth rather than a potential pathogen we are better able to model the changes in the gut ecosystem that are likely to occur in helminth therapy. McKenney et al. [30] previously showed that long term *H*. *diminuta* infection (continuous exposure beginning *in utero*) is associated with large compositional changes in the rat microbiota from Bacilli to Clostridia, but no change in alpha diversity.

Here, we assess the response of the gut microbiota and the host immune system to *H*. *diminuta* infection across a densely sampled time series spanning three months before and during infection. We ask (i) whether *H*. *diminuta* infection causes a disturbance of the gut microbiota in the prepatent or patent period and (ii) how long-term *H*. *diminuta* colonization influences the host immune system.

## Material and methods

### Animal use

**Fig 1:**
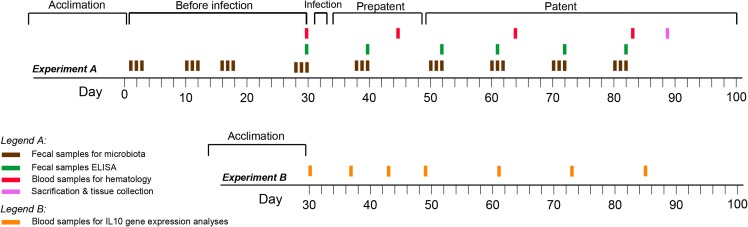
Experiment timeline. The entire experiment included five periods during which fecal, blood and tissue samples were collected: (i) acclimatization–rats were co-housed for a month prior to the start of experiment to acclimate laboratory conditions; (ii) before infection–the fecal samples for microbial analyses and for ELISA analyses were collected; (iii) infection–we infected rats in three consecutive days to prevent negativity of some animals from experimental group; (iv) prepatent period–period from infection to adults maturation, i.e. releasing of eggs in feces, (v) patent period–adults were present in the intestine; during prepatent and patent period were collected fecal, blood and tissue samples.

We performed two experiments, one in which we tracked the microbiota and broad immunological response (Experiment A), and a follow up in which we assessed IL-10 cytokine gene expression over time to characterize the type 2 immune response to *H*. *diminuta* infection (Experiment B; Fig 1). Each experiment was carried out with eight outbred female Wistar rats obtained when 20 weeks old and 240-270g from Charles River Laboratories (Sulzfeld, Germany; the supplier Velaz a.s., Prague, Czech Republic). Four were assigned to the experimental group and four to the negative control group. All rats were group-housed under a controlled temperature (22°C) and photoperiod (12:12-h light-dark cycle) and were provided unlimited access to rat chow and tap water. Rats were co-housed for a month prior to the start of the experiment to acclimate to laboratory conditions. During the experiment, rats were housed in pairs, with two cages for experimental and two cages for negative control rats.

The rats’ health, mortality and morbidity were recorded at 12-hour intervals. During this study, none of animals became ill, severely ill or died at any time prior to the experimental endpoint. All rats were euthanized by cervical dislocation, which is in accordance with the legislative regulations of the Czech government and European Union. The study was carried out in strict accordance with the recommendations in the Czech legislation (Act No. 166/1999 Coll., on veterinary care and on change of some related laws, and Act No. 246/1992 Coll., on the protection of animals against cruelty). The present experiments and protocols were approved by the Committee on the Ethics of Animals Experiments of the Biology Centre of the Czech Academy of Sciences (České Budějovice, Czech Republic; permit number: 1/2014) and by the Resort Committee of the Czech Academy of Sciences (Prague, Czech Republic).

### Culture of *Hymenolepis diminuta*, infectious doses and animal infections

*Hymenolepis diminuta* was cultured in the laboratory using grain beetles (*Tenebrio molitor*) as the intermediate host. Outbred rats, the natural definitive host, were used as the reservoir for *H*. *diminuta* adults under laboratory conditions. Grain beetles were fed rat feces containing *H*. *diminuta* eggs to establish the infection. Infectious doses of *H*. *diminuta* were prepared by dissecting the infectious stages, cysticercoids, from grain beetles. Each infectious dose contained approximately ten cysticercoids dissected directly from beetles under hygienic laboratory conditions. Each infectious dose was washed three times with sterile PBS (pH 7.4).

### Experimental setup

The Experiment A was carried out for 82 days, following a 30-day acclimation period, while the subsequent Experiment B was performed for the same period of *Hymenolepis* infection with a 7-day acclimation period (Fig 1). Within Experiment A, we collected several kinds of samples: (i) fecal samples and mucosal swabs for microbiota analysis, (ii) fecal samples for ELISA analysis of IgA antibodies to *H*. *diminuta*, (iii) blood samples for hematological analyses, and (iv) tissue samples for flow cytometry analysis of leukocyte cell numbers. In Experiment B, only blood samples for IL-10 gene expression were collected.

Fecal samples were collected over 30 days prior to *H*. *diminuta* infection to assess the baseline variability of the rat gut microbial community (Fig 1). Throughout the experiment, fecal samples were collected for microbial community analysis in bursts of three consecutive days followed by a seven to 10 day gap (Fig 1). Blood was collected for hematology analysis at four time points (days 29, 45, 71, 95), and feces were collected for IgA analysis at seven time points (Fig 1). Rats in the treatment group were infected with 10 cysticercoids via gavage per day on days 31, 32, and 33 to ensure infection. Negative control rats were gavaged with a PBS placebo using the same procedure. The rats in Experiment A were sacrificed on day 89, at which time the intestinal mucosa was swabbed for microbial analysis and intestinal and splenic tissue were collected for further analysis. Upon dissection we found that small intestine of each treated rat contained three to four *H*. *diminuta* adults. We note that the total helminth load of three to four adult *H*. *diminuta* per rat is likely similar to that in McKenney et al. [30] and is typical for *H*. *diminuta* in rats [32]. The blood samples for IL-10 analyses were collected in seven time points, on days 30, 37, 43, 49, 61, 73, and 85 (Fig 1).

We checked fecal samples of all rats for the presence of *H*. *diminuta* eggs daily beginning on day 45 (12 days post infection—dpi) using the modified Sheather flotation method (SpG 1.3) to confirm infection of rats in the treatment group and the absence of infection in the negative controls. All treatment rats started to shed eggs between day 47 and day 50 (16–19 dpi). Egg shedding means that mature adult worms were established in the rat and marks the beginning of the patent period. The prepatent period was recorded as the time between infection and the beginning of egg shedding (day 31 –day 50).

### Tissue collection and processing, leukocyte isolation and flow cytometry analyses

#### Blood collection

Blood samples were collected from ocular blood plexus in volume ca. 150–200 μl. For hematological analyses, the blood was placed to EDTA and shipped for hematological analyses to the clinical laboratory for small animals at Veterinary and Pharmaceutical University (Brno, Czech Republic). For cytokine analyses, the blood samples were processed as described below.

#### Intestinal and splenic tissue processing

The entire gastrointestinal tract and spleen were dissected out following sacrifice for activity of mucosal immunity analyzed using flow cytometry. The intestines were separated from the gut mesentery and the mesenteric lymph nodes. The intestines were divided into (i) duodenum + jejunum (the initial 40% of the small intestine), (ii) ileum (the latter 60% of the small intestine), and (iii) the entire colon. Intraepithelial leukocytes (IELs) and lamina propria leukocytes (LPLs) were isolated according to the following procedure. All intestinal parts were washed three times in sterile PBS (pH 7.4) and kept in 2% RPMI-1640 medium (Sigma-Aldrich, St. Louis, MO, USA) for 30 min at 4°C. They were then transferred into sterile tubes with 6 ml of HBSS (Thermo-Scientific, Waltham, MA, USA) and incubated in a water bath for 1 hr. at 37°C. The tissues were removed and the remaining suspension was centrifuged (10 min/150 g/4°C). The pellet was suspended in 5 ml of 30% Percoll (Sigma-Aldrich) and centrifuged (15 min/150 g/4°C). The pellet, remained after removing of supernatant, was mixed with 4 ml of 45% Percoll and overlaid with 3 ml 75% Percoll and centrifuged (30 min/150 g/4°C). The upper phase was removed and the layer of leukocytes on the lower phase was collected and washed twice in flow solution by centrifugation (10 min/150 g/4°C). The spleen was directly placed into chilled sterile 2% RPMI-1640 and passed through a plastic sieve with a sterile syringe plunger to isolate the splenocytes (SPLs). The resulting suspension was washed three times in 2% RPMI-1640 by centrifugation (10 min/150 g/4°C). Thereafter, the pellet was re-suspended in 1 mL of 10% RPMI-1640 (Sigma-Aldrich).

#### Leukocyte staining

The isolated leukocytes were transferred to the tubes for flow cytometry (Falcon-5ml Polystyrene Round Botton Tube; Corning Incorporated, Corning, NY, USA) and stained with a mixture of fluorescently labeled monoclonal antibodies. We optimized the dilution factor for each antibody (Table 1). We used three mixtures of monoclonal antibodies to observe specifically: leukocytes in general, natural killers, B-cells, and T-cells–Th-cells, Tc-cells, CD4/CD8 double negative T-cells (DN T-cells; γƍ-Tcells and other DN T-cells), and CD4/CD8 double-positive T-cells (DP T-cells; CD4+CD8αα and CD4CD8αβ). After 20 min of staining, each sample was washed with 2 ml of flow solution (1% FBS/PBS) by centrifugation (5 min/580 g/4°C). Then, the supernatant was discarded and the wet pellet was mixed with flow solution (0.5 ml for IELs+LPLs, and 1 ml for SPLs). The possibility of nonspecific antibody binding to the cell surface was removed by comparison with the unstained control samples, which was made from each sample of the spleen.

**Table 1:** List of anti-rat antibodies used in flow cytometry analyses.

Antibody name	Conjugate	Company
CD45 Mouse Anti-rat antibody (clone OX-1)	Pe-Cy®5.5	Life Technologies, Carlsbad, CA, USA
CD45R Mouse Anti-rat antibody (clone HIS24)	FITC	Life Technologies, Carlsbad, CA, USA
CD3 Mouse anti-rat antibody (Clone G4.18)	PerpCP-eFluor 710	Life Technologies, Carlsbad, CA, USA
CD4 Mouse Anti-rat	APC	Affymetrix eBiosciences, Hatfield, UK
CD8a Mouse Anti-rat PE-Cy7	Pe-Cy7	Affymetrix eBiosciences, Hatfield, UK
CD8b Mouse Anti-rat PE	PE	Affymetrix eBiosciences, Hatfield, UK
Anti-rat γƍ TCR antibody (clone V65)	FITC	Life Technologies, Carlsbad, CA, USA

#### Flow cytometry analyses

For obtaining of the exact numbers of leukocyte populations, we used the Flow-Count^TM^ Fluorospheres (Beckman Coulter, Brea, CA, USA). Each sample was measured using BD FACS CantoII flow cytometer (BD Biosciences, Franklin Lakes, NJ, USA; the device is equipped with two lasers: Coherent® Sapphire^TM^—Solid state, 488 nm—blue; and JDS Uniphase^TM^ HeNe—Air cooled, 633 nm—red) and always measured for 30,000 events. The resulting data were analyzed on BD FACSDiva software version 6.1.3 and converted from percent of cell population to absolute cell numbers in Excel MS Office version 8. We compared the cell numbers between the treatment group and control using two-sample t-tests in R. We report the uncorrected p-values for these tests because each test asks whether a certain cell type at a certain location is different between the control and infected groups, but each combination, and therefore each question, is unique.

### Detection of lumen IgA antibodies against *H*. *diminuta*

We analyzed the total concentration of IgA antibodies against *H*. *diminuta* in the rat intestine using an indirect ELISA.

#### Antigen preparation

We used crude *H*. *diminuta* antigen prepared from cysticercoids that were isolated from *Tenebrio molitor* to prevent cross-reaction against the rat gut microbiota. Ito et al. [36] revealed no difference in antigen specificity between cysticercoids and adults. *Hymenolepis* cysticercoids were dissected out of *T*. *molitor* and directly transferred to the solution of PBS (pH 7.4) in a sterile grinding mortar, homogenized with sterile pestle, sonicated, frozen, and the final supernatant was concentrated with Amicon ultra-0.5 centrifugal Filter 10 kDa (Merck Millipore, Billerica, MA, USA). Sonication was performed on sonicator Hielscher UP200S (Hielscher–Ultrasonics GmbH, Teltow, Germany) using a S3-microtip 3 needle under the conditions: two sonication cycles for 150s with 30 s cooling interval, 0.5 cycle, 20% amplitude. The freezing cycle was as follows: three cycles of freezing in liquid nitrogen (each for 4 min) interspersed with warming intervals in a thermostat at 45°C. The final crude antigen content was measured using Bradford assay with BSA as the standard.

#### Fecal extracts’ preparation and indirect ELISA

All fecal samples were weighed, mixed in 1 ml of PBS containing 1% FBS, centrifuged at 1,100 *g* for 10 min and the supernatants stored at –20°C until used. ELISA was performed with *H*. *diminuta* crude antigen preparation. Plates were coated using 10 μg of protein per well in 0.05M NaHCO_3_, pH 9.6, overnight at 4°C. To attain the same protein concentration in all fecal samples, we weighed each sample separately and recalculated the dilution of each sample based on the lowest recorded weight. Fecal extracts (1:800 in 0.05% Tween 20 in PBS) were incubated at RT for 1hr, washed, and incubated at RT for 1hr with peroxidase-conjugated goat anti-rat IgA (α-chain specific; Sigma-Aldrich) diluted 1:2000 in 0.05% Tween 20 in PBS. After a final wash, assays were developed with 0.1M phospho-citrate buffer, hydrogen peroxide and 2.5mM o-phenylenediamine (Sigma-Aldrich) and reactions were stopped with 2M sulfuric acid. Total concentration of IgA was measured using ELISA reader (Infinite® 200 Pro Series; Tecan, Maennedorf, Switzerland) at 490 nm and analyzed with Tecan i-control software (ver. 3.7.3.0). Absorbance values were compared with negative control samples and samples with absorbance greater than 0.6 were considered positive. Values for each time were compared with independent two-sample t-tests.

### Analyses of interleukin 10 gene expression

Total RNA from blood samples was extracted using HybridR Blood RNA Kit (GeneAll Biotechnology, Seoul, South Korea) and then reverse transcribed using High Capacity RNA-to-cDNA Kit (Thermo Fisher Scientific). Real-time PCR reactions were prepared using master-mix HOT FIREPol®Probe qPCR Mix Plus (Solis Biodyne, Tartu, Estonia) and specific probe and primers for interleukin 10 (IL-10; IL-10 Taqman gene expression assay for rats, Thermo Fisher Scientific) and analysed using Light Cycler LC480 (Roche, Basel, Switzerland). Relative expression of IL-10 was normalized to ubiquitin C (UBC—Ubc Taqman gene expression assay for rats, Thermo Fisher Scientific) using the mathematical model of Pfaffl [37]. Normalized Ct values were compared between uninfected and infected animals by Student’s t-test with unequal variance. Maximum normalization was used for visualization of IL-10 relative expression.

### Microbial DNA extraction, amplification, and analysis

#### DNA extraction and amplification

DNA was extracted from rat fecal samples at the Institute of Parasitology by first homogenizing with the FastPrep®-24 instrument (MP Biomedicals, Santa Ana, CA, USA). Second, DNA was purified using PSP® SPIN Stool DNA Plus Kit (Stratec Biomedical, Birkenfeld, Germany) according to the manufacturer’s protocol. For DNA amplification, we used primers and protocols modified from the Earth Microbiome Project. We used redesigned versions of the 515f/806r primers that target the V4 region of 16S rRNA in Bacteria and Archaea: 515f (5’–GTGYCAGCMGCCGCGGTAA–3’), which had 12nt Golay barcode and 806r (5’–GGACTACNVGGGTWTCTAAT–3’) (http://www.earthmicrobiome.org/emp-standard-protocols/16s/). Amplifications were conducted using 25μL reactions containing 5 Prime Hot Master Mix (5’–Item# 2200410) and 1 μL of genomic DNA. The PCR conditions were an initial denaturation step at 94°C for 3 minutes, followed by 25 cycles of 94°C for 45 seconds, 50°C for 60 seconds, and 72°C for 90 seconds, followed by a final extension step at 72°C for 10 minutes. PCR products were visualized on a gel and quantified using Picogreen (Thermofisher) according to the manufacturer’s protocol. Subsequently, 50 ng of each sample PCR product were pooled. The final pool was cleaned using the Ultraclean PCR cleanup kit (MO BIO Laboratories, Carlsbad, CA, USA) and sent for sequencing at the University of California in Los Angeles (Genoseq) sequencing core. The pool was sequenced on the Illumina MiSeq platform with paired end 2 x 250 sequencing and a separate 13-nucleotide index read.

#### Microbial data analysis

Data was retrieved from BaseSpace. Forward and reverse reads were trimmed to 200 bp using FastX Toolkit (http://hannonlab.cshl.edu/fastx_toolkit/index.html) to reduce the impact of low quality base reads. The size of the 16S amplicon is expected to be 300 bp. After trimming, the forward and reverse reads were joined and demultiplexed using QIIME version 1.9.0 [38] with scripts join_paired_ends.py and split_libraries_fastq.py using default parameters. This resulted in a total of nearly 13 million sequences. The sequence data and MiMARKs compliant metadata for this study are available at the European Bioinformatics Institute (accessions ERS1097065—ERS1097292).

#### Sequence clustering and phylogenetic tree assembly

Sequences were clustered into operational taxonomic units (OTUs) at 100% similarity (identical) using the Deblur denoising algorithm, which removes noise due to sequencing error [39]. Clusters of identical sequences allowed us to detect microbial changes at fine scale resolution. OTUs with fewer than five sequences were filtered out of the dataset resulting in a total of 9,135,369 sequences clustered into 1949 100% identity OTUs. Taxonomy was assigned in two steps. First, deblurred OTUs were clustered against the 99% identity Greengenes reference database and the 1349 OTUs that hit Greengenes at 100% identity were assigned the Greengenes OTU number and inherited Greengenes taxonomy, sequence, and position within the Greengenes reference tree. A phylogenetic tree was constructed by placing *de novo* reads within the Greengenes reference tree using the EPA algorithm within RAxML [40] following PyNAST alignment.

#### Microbial diversity analyses

Diversity analyses were conducted in QIIME and in Primer E [41]. The data were rarified to 7400 sequences per sample for analysis. Similarity in community composition (beta-diversity) was assessed with the Bray-Curtis dissimilarity metric, which takes into account shared OTUs and abundance [42], and with the UniFrac metric, which takes phylogenetic relationships into account [43]. Differences in community composition were visualized using Principle Coordinate analyses (PCoA) in Primer E [41] and in R using the Vegan package [44]. PERMANOVA tests conducted within Primer E were used to assess differences in community composition because they test for differences in the mean and are accurate even when dispersion is uneven for balanced designs such as the data here [45]. We tested for differences in dispersion across treatment groups separately using PERMDISP within Primer E. Community composition was more consistent within a single rat over the time than across rats, so rat is nested within treatment group for all PERMANOVAs.

We estimated bacterial diversity (richness) using the non-parametric Chao1 index chao [46] and implemented in QIIME. Chao1 estimates species abundance for each sample by adding a correction factor to the number of observed species in order to account for rare unsampled taxa, making it well suited for microbial datasets [47]. We compared Chao1 results to other alpha diversity metrics, including phylogenetic distance (PD_whole_tree) [48], and the Shannon index, all within QIIME. In order to determine the impact of *H*. *diminuta* on gut bacterial diversity we assessed alpha diversity changes over the time in the infected versus control group. There was not enough power to run a full mixed model analysis with rat as a random factor. Instead, we ran paired t-tests at each time point. Statistical analyses were conducted in the R environment for statistical computing v 3.0.1 [49].

We used DESeq2, which compares the distribution of count data across samples between groups [50], to identified taxa that were differentially enriched or depleted following *H*. *diminuta*. We required significantly associated OTUs to (i) differ significantly between infected and control treatment groups during the patent period, but not in the period before infection, and (ii) change significantly between the period before infection and the patent period in *H*. *diminuta*-treated rats. To be considered significant, we required these potential indicator OTUs to be present in more two or more rats that were not cage mates. Cage mates (Rat 1 and Rat 2; Rat 3 and Rat 4; Rat 5 and Rat 6; Rat 7 and Rat 8) shared a greater portion of the microbiota as a result of co-habitation.

## Results

### Changes in host immune response

#### Activity of intestinal and spleen immunity

**Fig 2:**
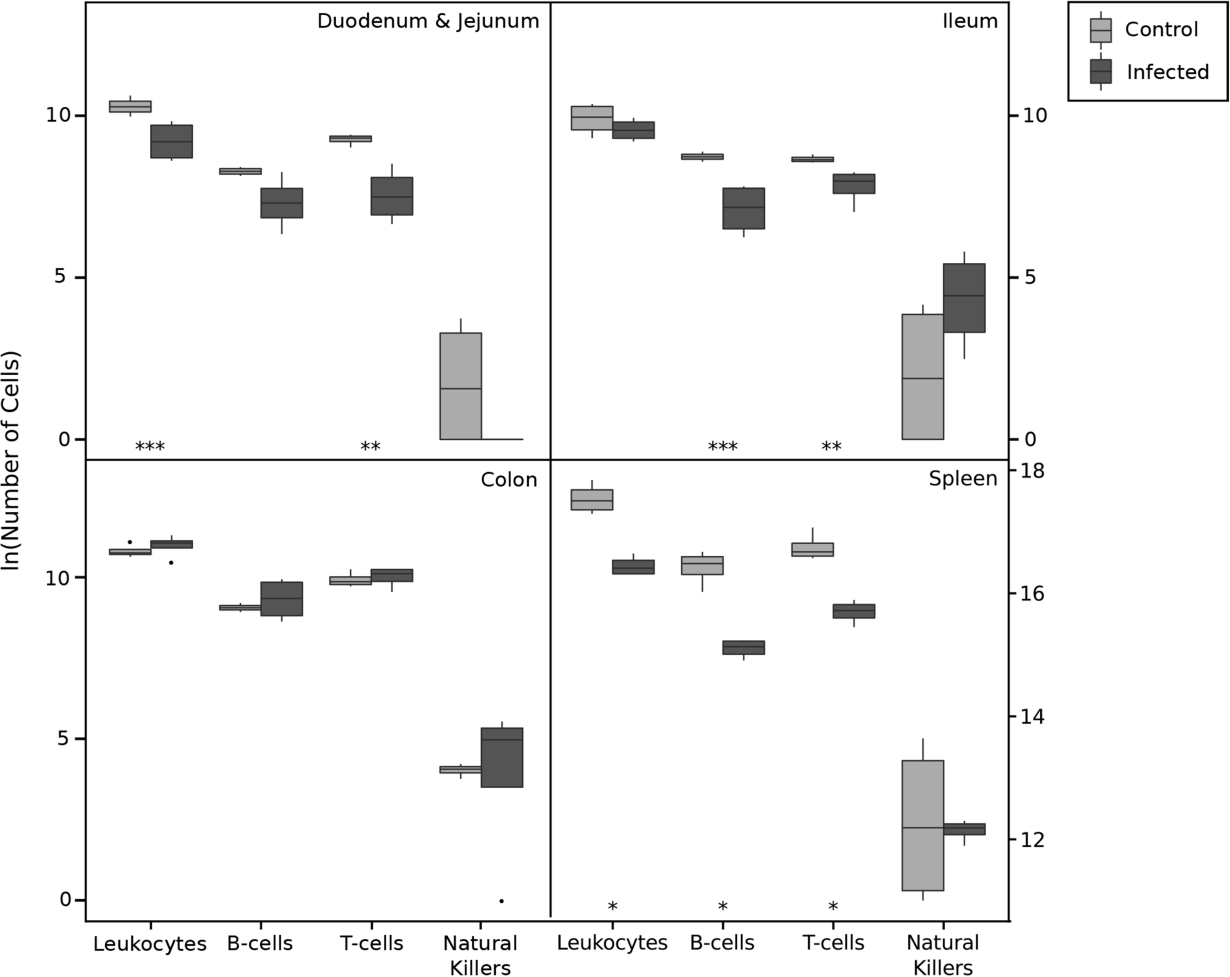
Flow cytometry boxplot panel figure—general leukocyte populations. Abundance of leukocyte populations in flow cytometric samples from rats infected with *Hymenolepis diminuta* (dark grey, n = 4) and control rats (light grey, n = 4). The error bars represent the 95% confidence intervals for the mean cell counts at each sample location for each group. The asterisks represent a significant difference in the mean cell counts at α = 0.05 level for t-tests implemented at each time point. (*): 0.01 ≤ p < 0.05, (**): 0.001< p < 0.01. [the numbers of cells are in the chart are given in 10^4^ in duodenum & ileum, colon, in case of spleen in 10^6^].

*Hymenolepis diminuta* induces changes in immune cell populations in rats, particularly in the numbers of B-cells and T-cells in the small intestine and spleen. Flow cytometry results show significant decreases in general leukocytes (CD45+), B-cell (CD45R+), and T-cell (CD3+) populations—but no change in natural killer (CD3-CD8a+) populations—in the small intestine (duodenum, jejunum and ileum) and spleen in *H*. *diminuta* infected rats (Fig 2). In contrast, leukocyte subpopulations in the colon were not significantly different from the negative controls (Fig 2). Within T-cell subpopulations, helper T-cell (Th- / CD4+) numbers declined in the small intestine and spleen, but increased in the colon (S1 Fig). Cytotoxic T-cells (Tc / CD8+) decrease in the spleen but do not change significantly otherwise (S1 Fig). Similarly, double negative T-cell DN- (CD4-CD8) populations as a whole and γδ T-cells and non-γδ DN T-cells, which include Tregs and dendritic cells, declined in the small intestine and spleen, but are unchanged in the colon (S2 Fig). CD4/CD8 double positive DP- (CD4+CD8+) T-cells, and ααT-cell and αβ T-cell subpopulations, all declined in the spleen (S3 Fig). ααT-cells declined in the small intestine, while αβ T-cells increased in the colon (S3 Fig). Overall, most immune cell populations are reduced in the spleen and small intestine, but are elevated or unchanged in the colon.

#### Hematological analyses

**Fig 3:**
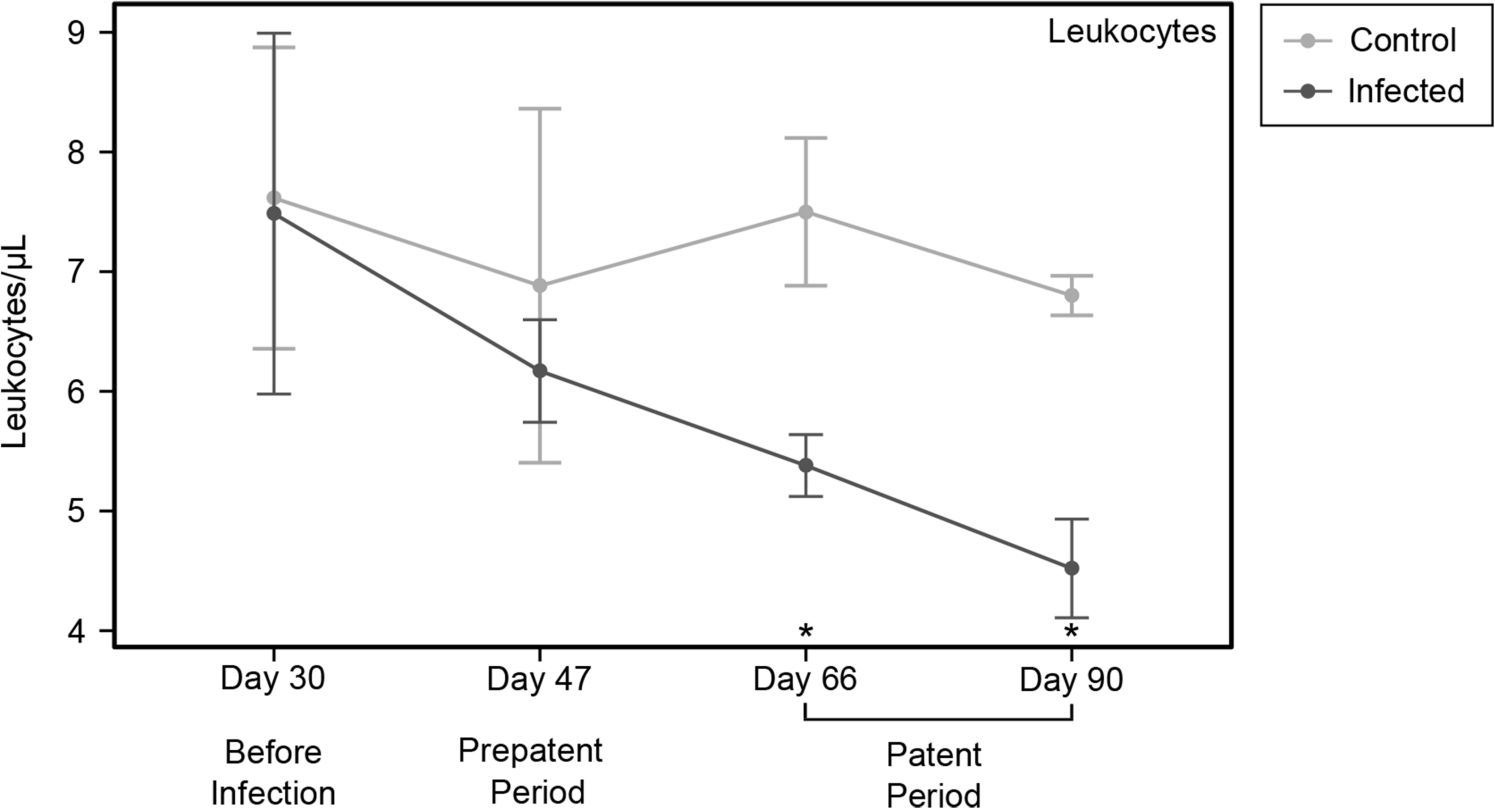
Hematology line graph panel figure for leukocytes. Abundance of leukocytes in blood samples of rats infected with *Hymenolepis diminuta* (dark grey, n = 4) and healthy, uninfected rats (light grey, n = 4). The samples were taken before infection, during the pre-patent period (after infection before establishment), and at two time points during the patent period (after the establishment of the infection). The error bars represent the 95% confidence interval of the mean cell counts for each group of rats at each time point. The asterisks represent a significant difference in the mean cell counts at α = 0.05 level for t-tests implemented at each time point. (*): 0.01 ≤ p < 0.05, (**): 0.001< p < 0.01.

We detected no change in hematological parameters over the course of *H*. *diminuta* infection, with the exception of leukocytes. Leukocytes decreased significantly in the rats treated with *H*. *diminuta* (Fig 3), which is concordant with the drop in leukocyte and spleenocyte populations recorded with flow cytometry data. Other cell types remained unchanged (S4–S6 Figs), including eosinophils (for details see S6 Fig).

#### Activity of IgA

**Fig 4:**
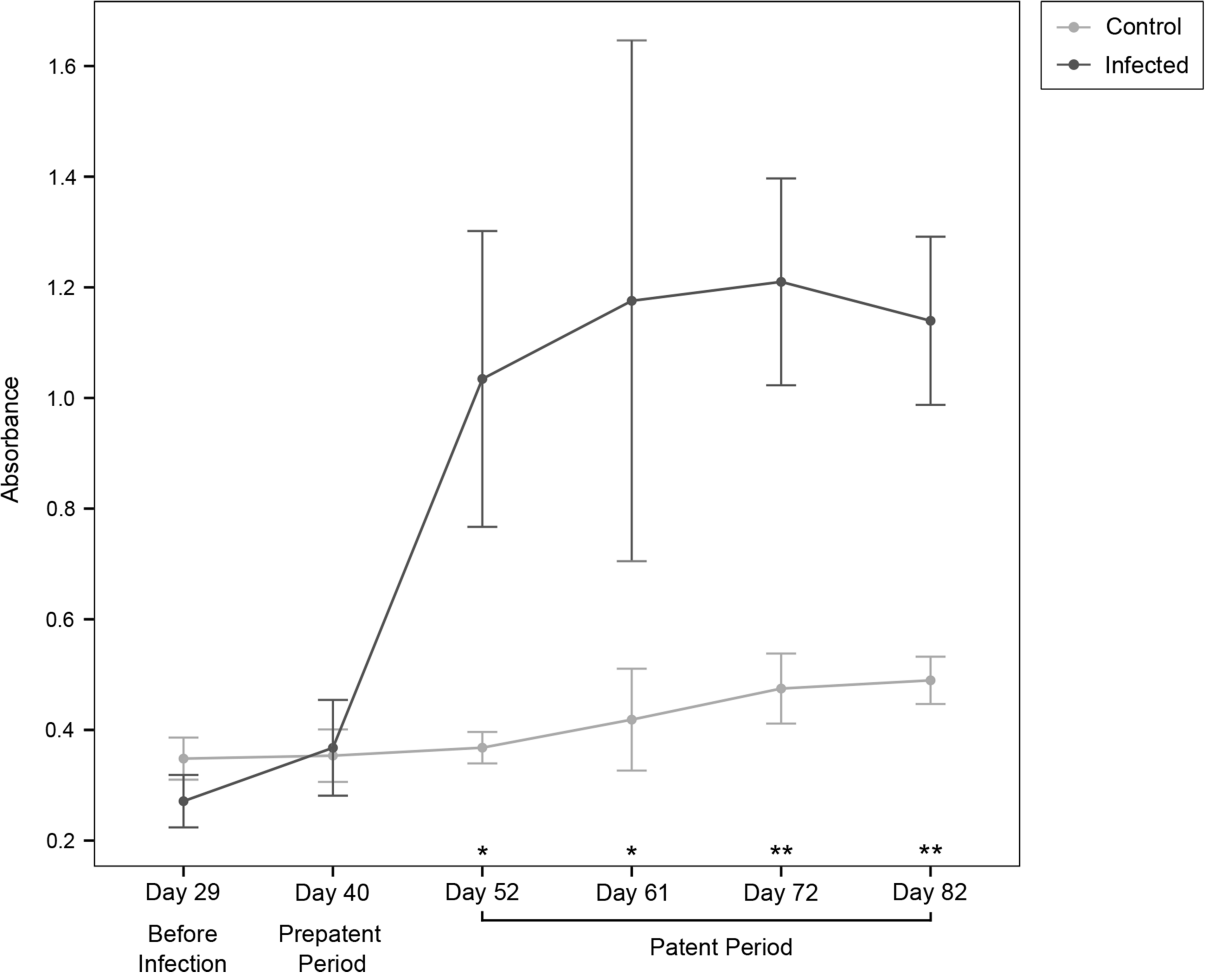
ELISA line graph for fluctuation of IgA antibodies from feces. Mean absorbance values for rats infected with *Hymenolepis diminuta* (dark grey, n = 4) and control rats (light grey, n = 4). Other notes as in Fig 3.

We determined the amount of IgA antibodies against *H*. *diminuta* present in the rat feces with ELISA analysis. We analyzed the fecal extracts from the *H*. *diminuta* infected group (n = 4 rats) and the control group (n = 4 rats) at six time points over the course of the experiment. Fecal samples were collected before infection (day 30), in the prepatent period (day 41) and in the patent period (days 53, 64, 73, and 83; for detail see Fig 1). Levels of IgA antibodies to *H*. *diminuta* only were significantly elevated in the *H*. *diminuta* infected group throughout the patent period (Fig 4).

#### Interleukin 10 expression during *H*. *diminuta* infection

**Fig 5:**
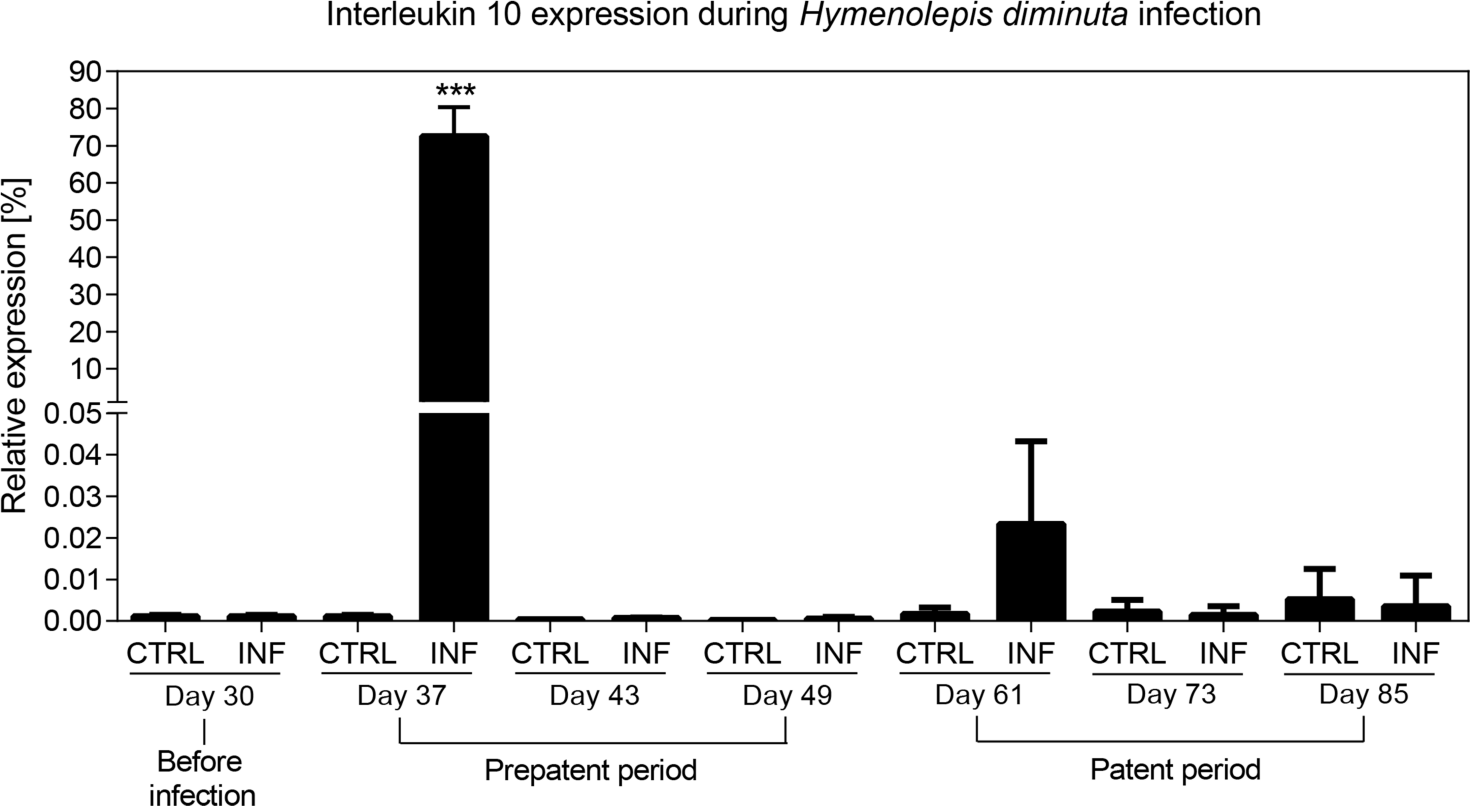
Relative expression of interleukin 10 during *Hymenolepis diminuta* infection. IL-10 gene expression in rat blood significantly increased during the early phase of prepatent period. In late prepatent period and patent period, expression of IL-10 dropped to basal levels. Expression is shown in relation to the UBC as the housekeeping gene. Error bars represent the standard errors from four independent biological replicates. (***): P<0.0001.

We assessed expression of the cytokine IL-10 using qPCR. In the *H*. *diminuta* infected group IL-10 expression is significantly elevated only in the early phase of prepatent period, i.e. six days post infection (Fig 5). There was no difference between *Hymenolepis*-treated and negative rats in the late prepatent period or patent period (Fig 5). In fact, expression of IL-10 had already dropped to basal levels at twelve days post infection (Day 43), which was the next time point measured (Fig 5).

### Gut microbiome diversity

#### Alpha diversity

**Fig 6:**
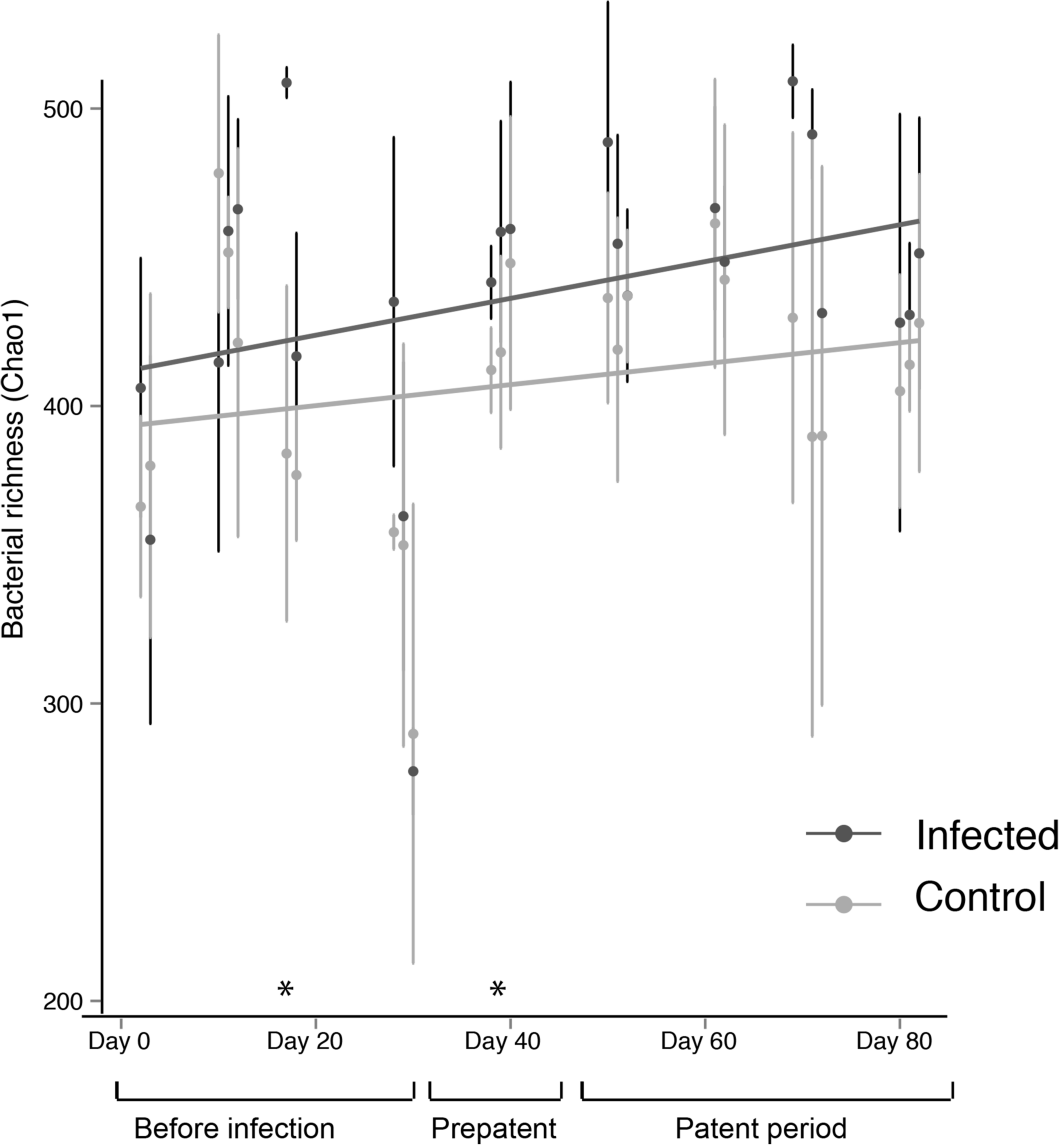
Alpha diversity of the microbiota does not change with *Hymenolepis diminuta* infection. Graph of Chao1 richness over the time. At each time point the average diversity and standard deviation are shown. Linear regression lines have been fitted to the mean to visualize the trend in diversity. The asterisks represent a significant difference in the mean diversity for two-sample t-tests implemented at each time point n = 4 for each treatment group at each time point.

*Hymenolepis diminuta* infection did not change alpha diversity in the gut microbiota. Diversity increased over the course of the experiment, but the increase was consistent across treatment groups (Fig 6). Throughout, the infected group harbored greater diversity, indicating that there were initial differences in alpha diversity, but the magnitude of the difference did not change following helminth introduction (Fig 6). The Chao [46] index of richness is shown, but these results hold for the Shannon index and diversity metrics that measure evenness and phylogenetic diversity.

#### Bacterial community composition

**Fig 7:**
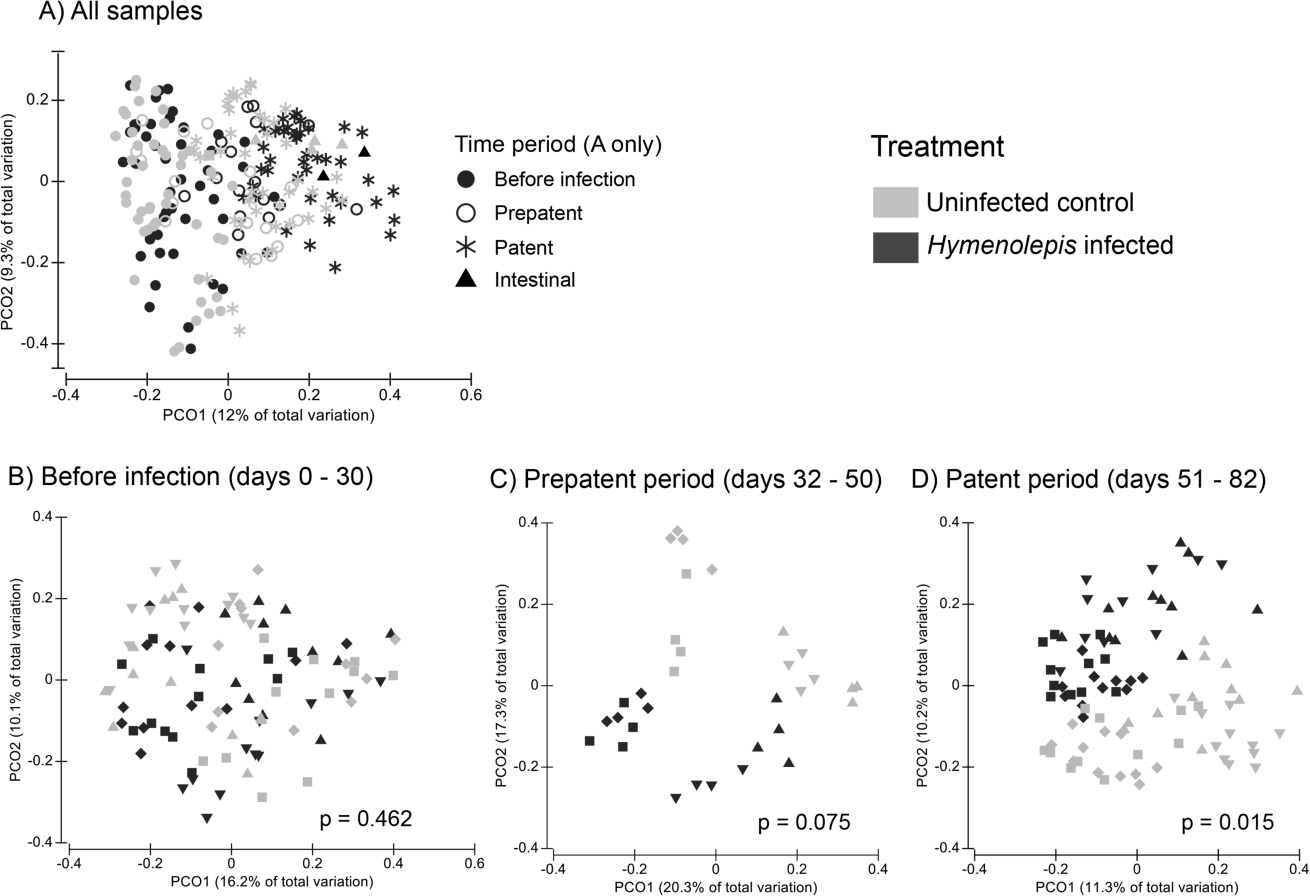
Composition of the gut microbiota shifted in response to *Hymenolepis diminuta* infection. Principle coordinate plots of the Bray-Curtis dissimilarity metric. A) All samples (N = 219) colored by treatment group and shaped according to time period. The seven samples are intestinal mucosa samples (triangles), and the rest are from feces. B-D) Fecal microbiota over the time course of infection. Gray = control and black = *H*. *diminuta* treatment. Each rat is represented by a different symbol/color combination. P-values for PERMANOVA with rat nested in treatment group are shown, and full results are in S1B Table) Before *H*. *diminuta* infection: Day 1 to Day 30 (n = 80), C) prepatent period of *H*. *diminuta* infection: Day 38 to Day 50 (n = 32). D) patent period of *H*. *diminuta* infection: Day 51 to Day 82 (n = 80).

Infection with *H*. *diminuta* induced significant shifts in the fecal bacterial community composition (beta diversity). We used a three factor PERMANOVA to assess differences between treatment groups, differences across time periods of infection, the interaction between these terms. We also included a term for rat nested within treatment group because each rat hosted a distinct microbiota (S1 Table). Bacterial community composition changed over time for both treatment groups (PERMANOVA time period: Pseudo-F = 4.05, df = 2, P = 0.001; treatment group: Pseudo-F = 2.625, df = 1, P = 0.001; Fig 7A). The interaction between time period and treatment group was not significant, see S1 Table for full model results. We also assessed treatment effects at different stages of infection taking rat into account with nested PERMANOVAs (Fig 7B–7D and S2 Table). There was no difference in community composition between treatment groups prior to infection or during the prepatent period (S2 Table; Fig 7B and 7C). The gut microbial communities are significantly different between treatment groups in the patent period (PERMANOVA: Pseudo-F = 2.24; df = 1; p = 0.015; Fig 7D) when *H*. *diminuta* was established in the small intestine and modulating the immune system. These results use the Bray Curtis dissimilarity index, which measure shared OTUs and includes abundance. We see similar results with the unweighted UniFrac metric, which takes into account phylogenetic relationships (S2 Table). However, there is no difference according to treatment group for the weighted UniFrac beta-diversity metric, which places a higher weight on common taxa (S2 Table). This suggests that rare taxa may account for the differences between the treatment groups. Most of our data come from fecal samples, but intestinal samples were taken from four of the eight rats in the experiment, and three additional uninfected rats. Intestinal sample sizes are too small for statistical analysis, but they roughly cluster according to time period and treatment group (Fig 7A).

#### Taxonomic composition of the rat microbiome

**Fig 8:**
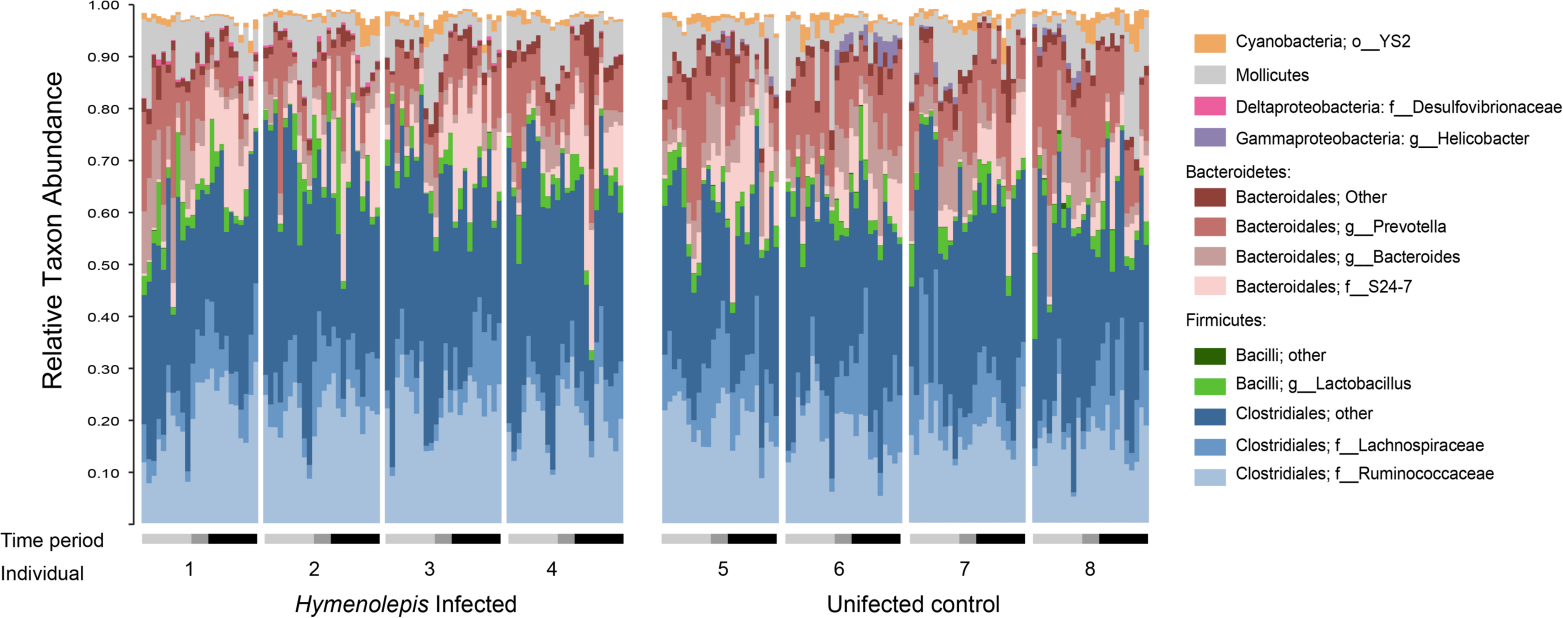
Taxonomic composition at the genus level is largely unchanged following *Hymenolepis* infection. Relative abundance of dominant taxa. Bars do not add up to 100% because rare taxa are not shown. “Closteriales; Other” represents all OTUs within Clostridales except those in the Ruminococcaceae. “Bacteroidales; Other” represents all OTUs within Bacteroidales except those in the family S24 and the genera *Prevotella* and *Bacteroides*. “Bacilli; Other” represents all OTUs within Bacilli except those in *Lactobacillus*. Infection period: light gray = before infection (n = 10 per rat), gray = prepatent period (n = 4 per rat), black = patent period (n = 10 per rat).

At a broad level, members of the Bacteroidetes, Firmicutes, and Mollicutes that are characteristic of rodent gut microbiota generally dominated the rat microbiota here. Taxonomic composition at the genus level was consistent across treatment groups and largely stable over the time (Fig 8). However, ordination plots (Fig 7) showed that community composition did change, so we sought to identify taxa that specifically responded to *H*. *diminuta* infection at the strain and OTU level using DESeq2 [50]. Nearly all of the 48 OTUs that change significantly in response to *H*. *diminuta* infection belong to species- or genus-level taxa that are abundant in both treatment groups (S3 Table). For example, several OTUs within the uncultured Bacteroidales family S24-7 and Ruminococcaceae, and Mollicutes order RF39 were significantly enriched in *H*. *diminuta* treatments. Different OTUs in each of these groups were also significantly enriched in the control rats, as were *Turcibacter*, Erysipelotrichaceae, and *Sutterella*. Previous studies have found that helminth infection significantly increases *Lactobacillus* in the mouse microbiome [18], but we did not find such association here (S7C Fig).

## Discussion

Helminths modulate the mammalian immune system, and as a result they are candidates for therapeutic agents in the treatment and prevention of immune-mediated inflammatory diseases (IMIDs). To date, clinical trials of helminth therapy have had mixed success [51]. The microbiota constitute a major part of the gut ecosystem [12], and are also implicated in IMIDs development [13]. There is growing evidence that helminth infection alters the gut microbiota in a range of host species, including humans, pigs, macaques, mice, and rats (reviewed in [18]), and indeed, helminth-induced changes in the microbiota directly contribute to protection from IMIDs [21, 22, 52].

The microbial responses to helminths vary widely across systems, and run the gamut from inducing dysbiosis and lower diversity [24, 25], to causing no impact [28, 53] to restoring a healthy microbial community and increasing diversity [26, 29]. These diverse responses highlight the need to investigate diverse helminths in order to build a broad understanding their overall impact on the gut ecosystem, and how this varies across helminth species that differ in pathogenicity and other aspects of their biology.

We monitored changes in the rat gut microbiota over the course of infection with the benign tapeworm *H*. *diminuta*. Alpha diversity did not change in the response to *H*. *diminuta* infection (Fig 6), consistent with the previous study of *H*. *diminuta* in rats [30]. By observing the microbiota over time we show that there is no short-term response to *H*. *diminuta* infection followed by recovery of the community, but instead alpha diversity increases fairly steadily over time in both treatment groups as the rats mature (Fig 6). In contrast, studies in mouse models typically find significant decreases in alpha diversity of the gut microbiota after helminth infection [24, 25]. Decreasing alpha diversity is a sign of dysbiosis in many human diseases and in other systems [19]. Thus, the drop in microbiota diversity in mice but not rats may reflect the pathogenic nature of *H*. *diminuta* infection in mice but benign infection in rats.

Though alpha diversity is stable, we do see a shift in microbiota community composition (i.e., beta diversity) in the rats after infection with *H*. *diminuta* (Figs 7 and 8). We see no difference in the community composition between control group and *H*. *diminuta*-treated group before infection (Fig 7B), but the groups were significantly different by the patent period (i.e., the time when adult are developed and releasing eggs; Fig 7D). Tracking microbiota over time points to a gradual shift in bacterial composition and complements previous work showing compositional shifts after long-term infection (beginning in utero) with *H*. *diminuta* is associated with compositional changes in the rat gut microbiota [30]. We find that the compositional shift at the community level (beta diversity) is a result of changes at the strain and species level (Fig 8 and S3 Table) rather than broad shifts from Bacilli (Firmicutes) to Clostridia (Bacteroidetes) observed previously in *H*. *diminuta* infected rats [30] (S7 Fig). Studies in mice have documented broad taxonomic shifts as a result of helminth infection, such as decreased Bacteroidetes diversity [24] and increased relative abundance of *Lactobacillus* [18, 24], that are not observed here (Fig 8 and S7 Fig). The potential functional importance of the fine-scale community shifts here or more substantial changes reported by McKenney et al. [30] is unclear. However, the taxa involved and the maintenance of alpha diversity suggest that *H*. *diminuta* does not induce dysbiosis in rats.

Our results suggest that *H*. *diminuta* induces a type 2 immune response and an immunoregulatory response consistent with previous studies of *H*. *diminuta* [33], and characteristic of helminths infections generally [35]. Interleukin 10 (IL-10) expression peaked during the prepatent period, measured at seven days post infection, when larval stages are present (Fig 5). Strong type 2 immune responses contribute to expulsion of the larval worms during the prepatent period in mice, a non-permissive host [33]. *Hymenolepis diminuta* establishes long-term infections in rats, but only a handful of adults are tolerated [32, 34]. Thus, the elevation of IL-10 in the prepatent period detected here, and inferred type 2 immune response, might be caused by the invasion of larval stages into gut tissues as described previously [54, 55], or expulsion of many immature worms via a similar type 2 immune response [33, 34]. Consistent with the latter scenario, we administered 30 cysticercoids and find only 3–4 adults of *H*. *diminuta* per rat at 60 days post infection. However, further experiments tracking the type 2 cytokines, cellular immunity, and worm load over the initial period of infection are necessary to establish the dynamics of the early immune response in rats.

We saw elevated IgA antibodies in the feces directed against *H*. *diminuta* throughout patent period when adult tapeworms are established (Fig 4), a sign of activation of mucosal immunity. We do not see larger B-cell populations in *H*. *diminuta* treated rats (Fig 2), though this is likely because we only measured the general B-cell population (CD45R+) and could not specifically detect activated IgA^+^ B cells or IgA-producing plasmablasts [56]. Further research is necessary.

Using flow cytometry to assess immune cell populations from the small intestine, colon, and spleen we observed immunomodulation in the patent period of *H*. *diminuta* infection in rats. In the spleen and small intestine—the localization site of *H*. *diminuta* adults—we found significantly fewer leukocytes, and T-cells and B-cells specifically, in the presence of *H*. *diminuta* (Fig 2). We also see suppression of most T-cell subpopulations in the spleen and to a lesser extent in the intestine (S1–S3 Figs). The number of leukocytes detected in peripheral blood by hematological analysis is significantly lower in the patent period in *H*. *diminuta* infected rats (Fig 3). Overall leukocyte numbers did not change in the colon (Fig 2). Helper Th2-cells largely govern the immune response against helminths generally, and against *H*. *diminuta* in particular both in rats and in non-permissive mouse hosts [34, 57– 59]. Here we see significant reduction in helper T-cells in the spleen in the presence of *H*. *diminuta*, but an increase in the colon (S1 Fig). These data suggest that the *H*. *diminuta* adults suppress the proliferation of leukocytes, including most populations of T-cells, but we cannot rule out the possibility that *H*. *diminuta* induces T-cell migration to other parts of the body, e.g. the mesenteric lymph nodes. Many helminths, such as *Heligmosomoides polygyrus*, *Taenia crassiceps* and *Echinococcus granulosus*, modulate and suppress the immune system and can block T-cell proliferation [1]. In *H*. *diminuta*, adult worms produce an excretory or secretory product (*Hd*HMW) that inhibits the T-cell proliferation *in vitro* without cytotoxic effect [33, 60], and reduced inflammatory disease after intraperitoneal injection to mice with induced colitis [58]. We cannot comment on Tregs, as they are included in the group of double-negative T-cells but not specifically measured.

Our hematological data showed no eosinophilia in rats over the course of *H*. *diminuta* infection (S6 Fig), which is an expected part of the type 2 immune response to *H*. *diminuta* in mice [33, 57] and rats [34]. However, this requires further study as Goswami et al. [61] rarely found tissue eosinophilia in *H*. *diminuta* infected rats, and peripheral blood may not be suitable for characterizing eosinophilia.

Overall, our immunological data suggest that *H*. *diminuta* larvae induce a short type 2 immune response in the very beginning of infection—prepatent period—that likely contributes to expulsion of many worms. Later, during the patent period, established adults modulate the immune system to prevent their expulsion from the host organism.

## Conclusion

*Hymenolepis diminuta* infection in rats is a model system for investigating the impact of a benign helminth on the gut ecosystem, including the microbiota and immune system, and a candidate therapeutic helminth for human immune-mediated diseases. *Hymenolepis diminuta* induces a type 2 immune response coupled with immune regulation that is typical of helminth infections more broadly. We show that the composition of the microbiota changes over the course of infection, but changes are subtle. We see no change in alpha diversity with *H*. *diminuta* infection and fine-scale changes in composition. The unchanged alpha diversity and absence of dysbiosis are consistent with previous study of *H*. *diminuta* in rats, but contrary to most pathogenic helminths in mice. We do not find evidence of microbiota disturbance early in infection when the larvae are more immunogenic. These results suggest that *H*. *diminuta* could be used therapeutically without disrupting the gut ecosystem—at least in rats.

## Supporting information

S1 Fig Flow cytometry boxplot panel figure—T-cell populations. Abundance of T-cell populations in flow cytometric samples from rats infected with *Hymenolepis diminuta* (dark grey, n = 4) and uninfected rats (light grey, n = 4). The error bars represent the 95% confidence intervals for the mean cell counts at each sample location for each group. The asterisks represent a significant difference in the mean cell counts at the α = 0.05 level for t-tests implemented at each time point. (*): 0.01 ≤ p < 0.05, (**): 0.001< p < 0.01. [the numbers of cells are in the chart are given in 10^4^ in duodenum & ileum, colon, in case of spleen in 10^6^].
(PDF)

S2 Fig Flow cytometry boxplot panel figure—double negative T-cell populations. Abundance of double-negative T-cell populations in flow cytometric samples from rats infected with *Hymenolepis diminuta* (dark grey, n = 4) and uninfected rats (light grey, n = 4). The error bars represent the 95% confidence intervals for the mean leukocyte cell counts at each sampling location for each group of rats. The asterisks represent a significant difference in the mean cell counts at the α = 0.05 level for t-tests implemented at each time point. (*): 0.01 ≤ p < 0.05, (**): 0.001< p < 0.01. [the numbers of cells are in the chart are given in 10^4^ in duodenum & ileum, colon, in case of spleen in 10^6^].
(PDF)

S3 Fig Flow cytometry boxplot panel figure—double positive T-cell populations. Abundance of double-positive T-cell populations in flow cytometric samples from rats infected with *Hymenolepis diminuta* (dark grey, n = 4) and uninfected rats (light grey, n = 4). The error bars represent the 95% confidence intervals for the mean leukocyte cell counts at each sampling location for each group of rats. The asterisks represent a significant difference in the mean cell counts at the α = 0.05 level for t-tests implemented at each time point. (*): 0.01 ≤ p < 0.05, (**): 0.001< p < 0.01. [the numbers of cells are in the chart are given in 10^4^ in duodenum & ileum, colon, in case of spleen in 10^6^].
(PDF)

S4 Fig Hematology line graph panel–red blood cells. Abundance of red blood cells, hemoglobin and hematocrit in blood samples of rats infected with *Hymenolepis diminuta* (dark grey, n = 4) and healthy, uninfected rats (light grey, n = 4). The samples were taken before infection, during the pre-patent period (after infection before establishment), and at two time points during the patent period (after the establishment of the infection). The error bars represent the 95% confidence interval of the mean cell counts for each group of rats at each time point. The asterisks represent a significant difference in the mean cell counts at α = 0.05 level for t-tests implemented at each time point. (*): 0.01 ≤ p < 0.05, (**): 0.001< p < 0.01.
(PDF)

S5 Fig Hematology line graph panel–red blood cells indices. Abundance of red blood cells indices–MCH, MCHC and MCV in blood samples of rats infected with *Hymenolepis diminuta* (dark grey, n = 4) and healthy, uninfected rats (light grey, n = 4). The samples were taken before infection, during the pre-patent period (after infection before establishment), and at two time points during the patent period (after the establishment of the infection). The error bars represent the 95% confidence interval of the mean cell counts for each group of rats at each time point. The asterisks represent a significant difference in the mean cell counts at α = 0.05 level for t-tests implemented at each time point. (*): 0.01 ≤ p < 0.05, (**): 0.001< p < 0.01.
(PDF)

S6 Fig Hematology line graph panel–specific leukocyte populations. Abundance of specific leukocyte populations (basophils, eosinophils, lymphocytes, monocytes and neutrophils) in blood samples of rats infected with *Hymenolepis diminuta* (dark grey, n = 4) and healthy, uninfected rats (light grey, n = 4). The samples were taken before infection, during the pre-patent period (after infection before establishment), and at two time points during the patent period (after the establishment of the infection). The error bars represent the 95% confidence interval of the mean cell counts for each group of rats at each time point. The asterisks represent a significant difference in the mean cell counts at α = 0.05 level for t-tests implemented at each time point. (*): 0.01 ≤ p < 0.05, (**): 0.001< p < 0.01.
(PDF)

S7 Fig Phylum specific analyses. Before infection includes 12 time points, prepatent period includes 4 time points, and patent period includes 11 time points. A and B) Alpha diversity, as measured by the Shannon diversity index, of Bacteroidetes and Firmicutes phyla. A) Shannon diversity within the Bacteroidetes phylum increases in response to helminth infection, but the effect is not significant. B) There is no response in Shannon diversity of the Firmicutes following helminth introduction. C) The relative abundance of the genus *Lactobacillus* does not change following helminth introduction.
(PDF)

S1 Table Statistical similarity in community composition (beta diversity). Three-factor PERMANOVA with time period, treatment group, and rat nested within treatment group. Model includes interaction between treatment group and time period. Test used unrestricted permutation of raw data and Type III sum of squares. Bray-Curtis dissimilarity metric.
(DOCX)

S2 Table Comparison of community composition within time periods. Nested PERMANOVA analyses (rat nested within treatment group) were run to differences in community composition similarity before infection (12 time points), in the prepatent period (4 time points), and in the patent period (11 time points). Beta diversity metrics: UW–unweighted UniFrac, W–weighted UniFrac, BC–Bray Curtis). Test used unrestricted permutation of raw data and Type III sum of squares. PERMDISP was used to assess differences in dispersion between treatment groups.
(DOCX)

S3 Table OTU table highlighting changes in response to helminth infection. OTUs are identical sequences (100% similarity). Bonferroni corrected Kruskal Wallis test results are shown comparing 1) samples from the time period before infection to the patent period and 2) comparing samples from the infected versus control group all within the patent period. Relative abundance of each OTU is shown for these comparison groups. Absolute sequence counts are shown for each rat summed across all 11 samples of the patent period. Potential indicators of helminth infection are highlighted in gray. *Note–S3 Table is in excel file*.
(XLS)
