# Supplementary figures and images for: A benign helminth alters the host immune system and the gut microbiota in a rat model system
Source: PLoS One. 2017 Aug 3;12(8):e0182205. doi: 10.1371/journal.pone.0182205 (PMC5542714; doi:10.1371/journal.pone.0182205)

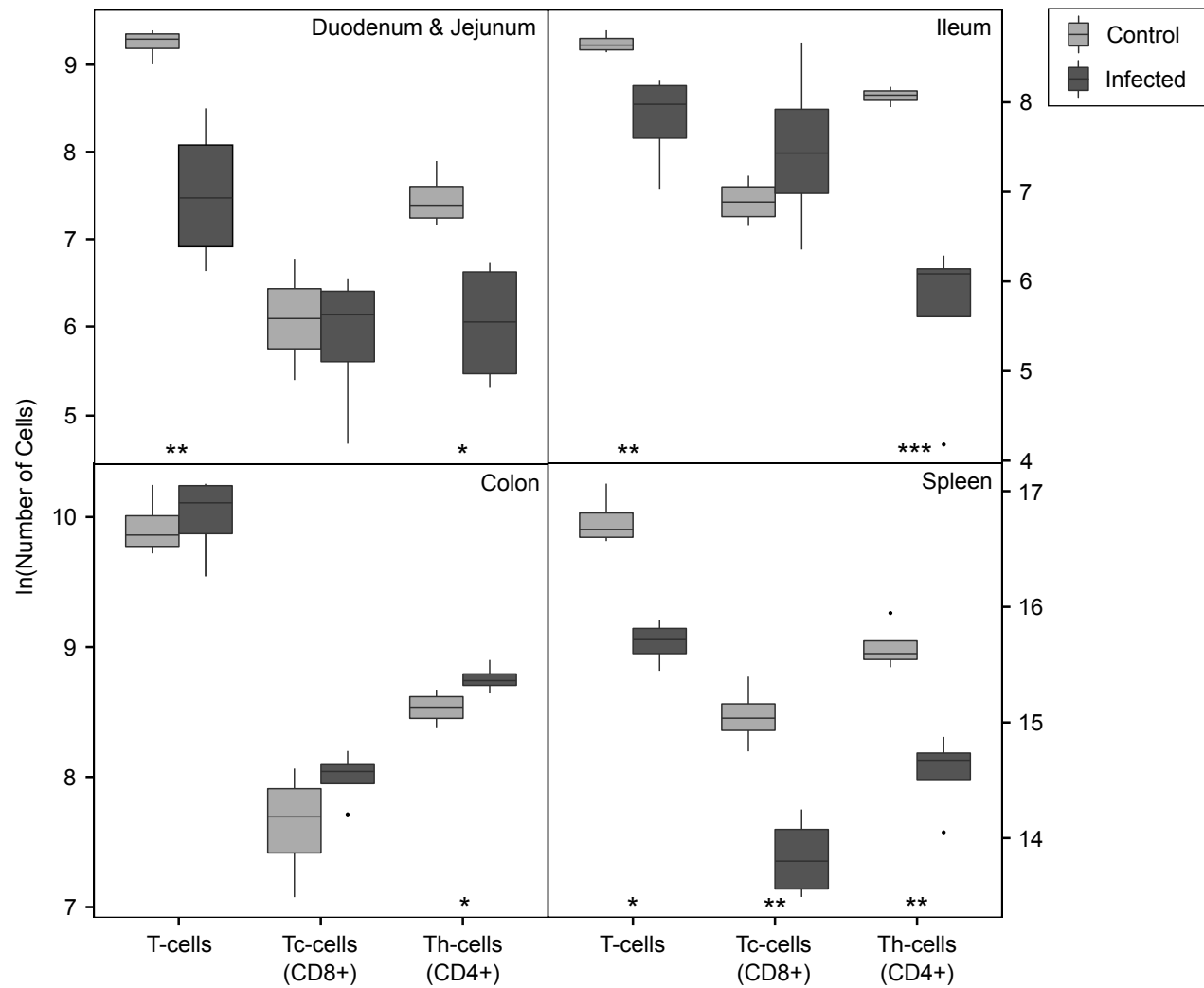

Supplement: S1 Fig — Abundance of T-cell populations in flow cytometric samples from rats infected with Hymenolepis diminuta (dark grey, n = 4) and uninfected rats (light grey, n = 4). The error bars represent the 95% confidence intervals for the mean cell counts at each sample location for each group. The asterisks represent a significant difference in the mean cell counts at the α = 0.05 level for t-tests implemented at each time point. (*): 0.01 ≤ p < 0.05, (**): 0.001< p < 0.01. [the numbers of cells are in the chart are given in 104 in duodenum & ileum, colon, in case of spleen in 106]. (PDF) [file pone.0182205.s001.pdf]

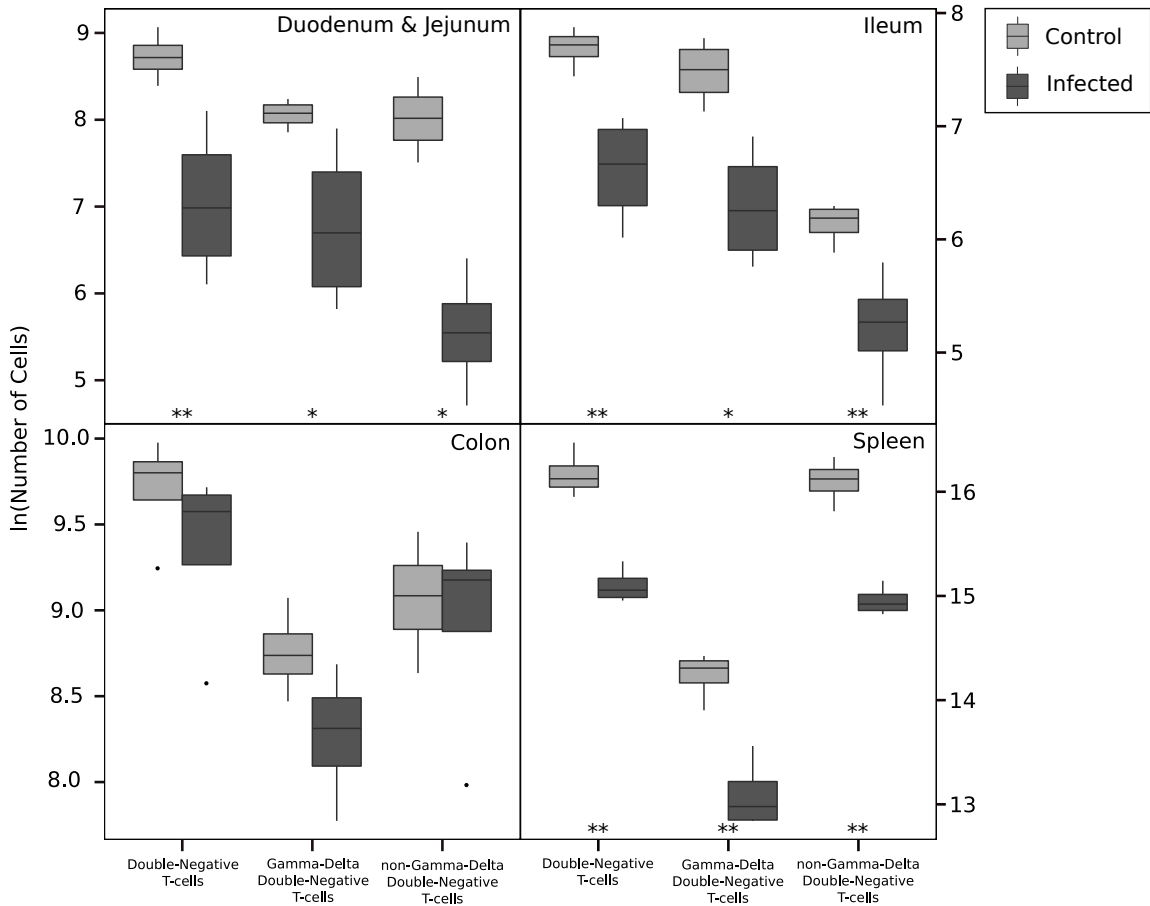

Supplement: S2 Fig — Abundance of double-negative T-cell populations in flow cytometric samples from rats infected with Hymenolepis diminuta (dark grey, n = 4) and uninfected rats (light grey, n = 4). The error bars represent the 95% confidence intervals for the mean leukocyte cell counts at each sampling location for each group of rats. The asterisks represent a significant difference in the mean cell counts at the α = 0.05 level for t-tests implemented at each time point. (*): 0.01 ≤ p < 0.05, (**): 0.001< p < 0.01. [the numbers of cells are in the chart are given in 104 in duodenum & ileum, colon, in case of spleen in 106]. (PDF) [file pone.0182205.s002.pdf]

In(Number of Cells)

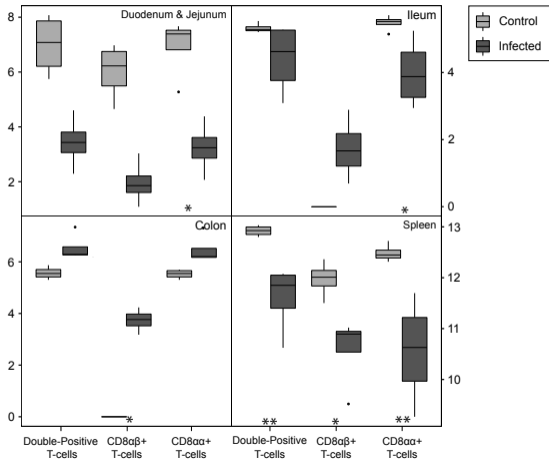

Supplement: S3 Fig — Abundance of double-positive T-cell populations in flow cytometric samples from rats infected with Hymenolepis diminuta (dark grey, n = 4) and uninfected rats (light grey, n = 4). The error bars represent the 95% confidence intervals for the mean leukocyte cell counts at each sampling location for each group of rats. The asterisks represent a significant difference in the mean cell counts at the α = 0.05 level for t-tests implemented at each time point. (*): 0.01 ≤ p < 0.05, (**): 0.001< p < 0.01. [the numbers of cells are in the chart are given in 104 in duodenum & ileum, colon, in case of spleen in 106]. (PDF) [file pone.0182205.s003.pdf]

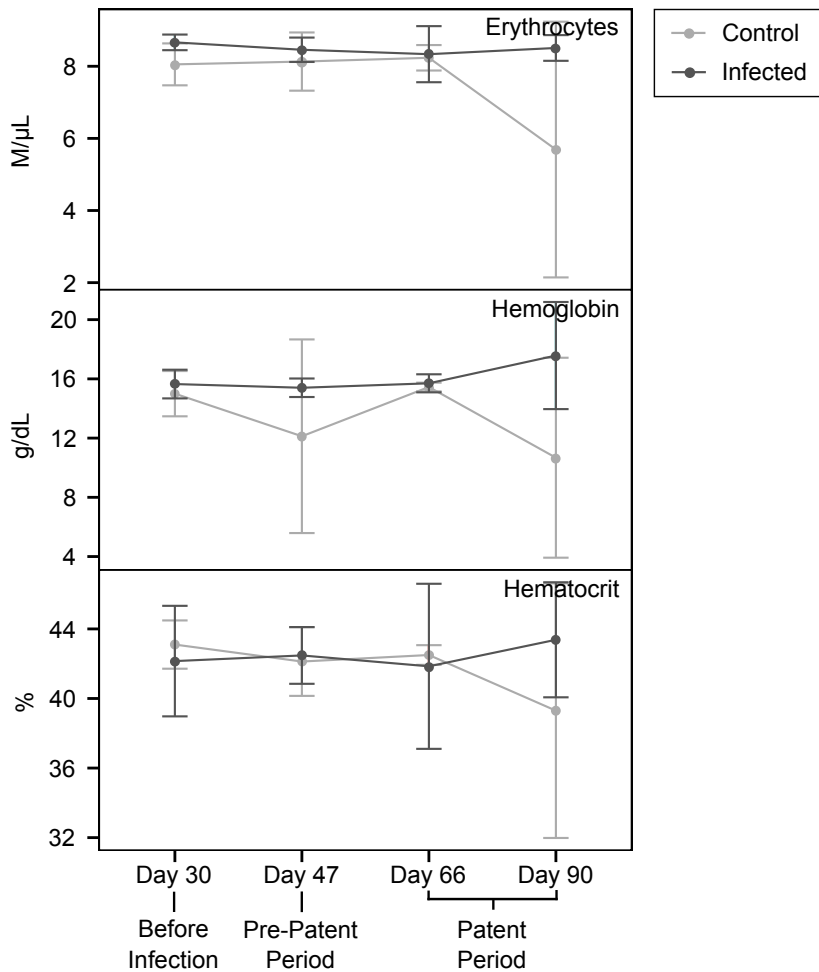

Supplement: S4 Fig — Abundance of red blood cells, hemoglobin and hematocrit in blood samples of rats infected with Hymenolepis diminuta (dark grey, n = 4) and healthy, uninfected rats (light grey, n = 4). The samples were taken before infection, during the pre-patent period (after infection before establishment), and at two time points during the patent period (after the establishment of the infection). The error bars represent the 95% confidence interval of the mean cell counts for each group of rats at each time point. The asterisks represent a significant difference in the mean cell counts at α = 0.05 level for t-tests implemented at each time point. (*): 0.01 ≤ p < 0.05, (**): 0.001< p < 0.01. (PDF) [file pone.0182205.s004.pdf]

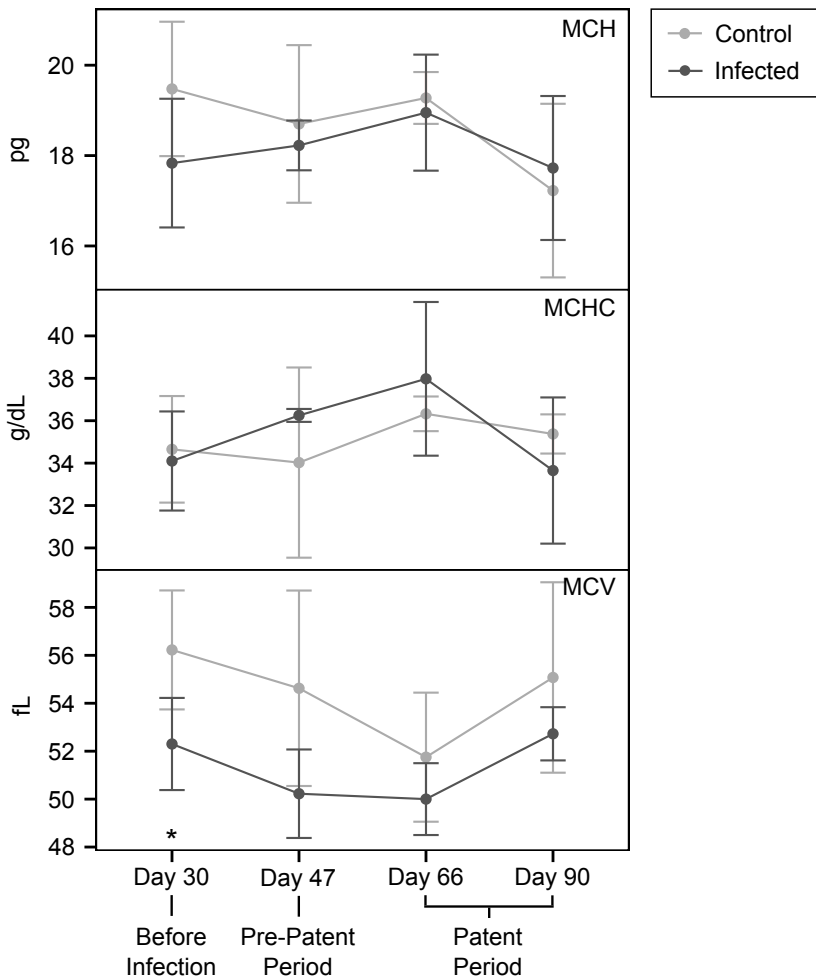

Supplement: S5 Fig — Abundance of red blood cells indices–MCH, MCHC and MCV in blood samples of rats infected with Hymenolepis diminuta (dark grey, n = 4) and healthy, uninfected rats (light grey, n = 4). The samples were taken before infection, during the pre-patent period (after infection before establishment), and at two time points during the patent period (after the establishment of the infection). The error bars represent the 95% confidence interval of the mean cell counts for each group of rats at each time point. The asterisks represent a significant difference in the mean cell counts at α = 0.05 level for t-tests implemented at each time point. (*): 0.01 ≤ p < 0.05, (**): 0.001< p < 0.01. (PDF) [file pone.0182205.s005.pdf]

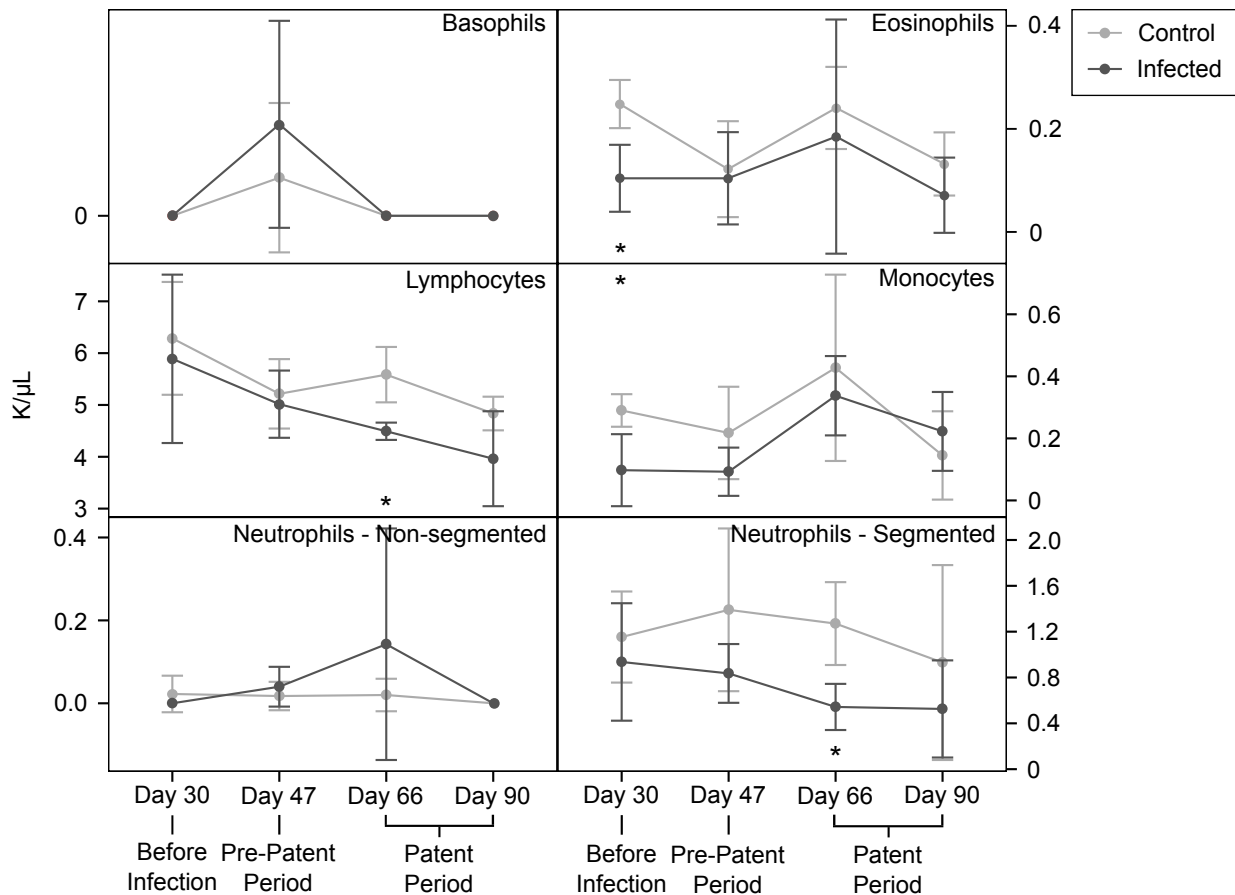

Supplement: S6 Fig — Abundance of specific leukocyte populations (basophils, eosinophils, lymphocytes, monocytes and neutrophils) in blood samples of rats infected with Hymenolepis diminuta (dark grey, n = 4) and healthy, uninfected rats (light grey, n = 4). The samples were taken before infection, during the pre-patent period (after infection before establishment), and at two time points during the patent period (after the establishment of the infection). The error bars represent the 95% confidence interval of the mean cell counts for each group of rats at each time point. The asterisks represent a significant difference in the mean cell counts at α = 0.05 level for t-tests implemented at each time point. (*): 0.01 ≤ p < 0.05, (**): 0.001< p < 0.01. (PDF) [file pone.0182205.s006.pdf]

A) Bacteroidetes diversity

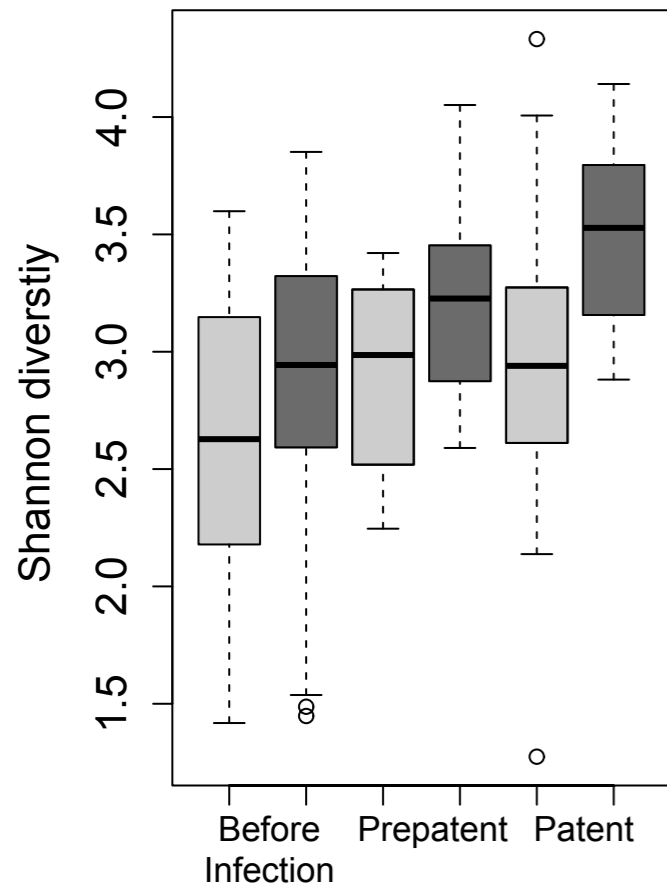

B) Firmicutes diversity

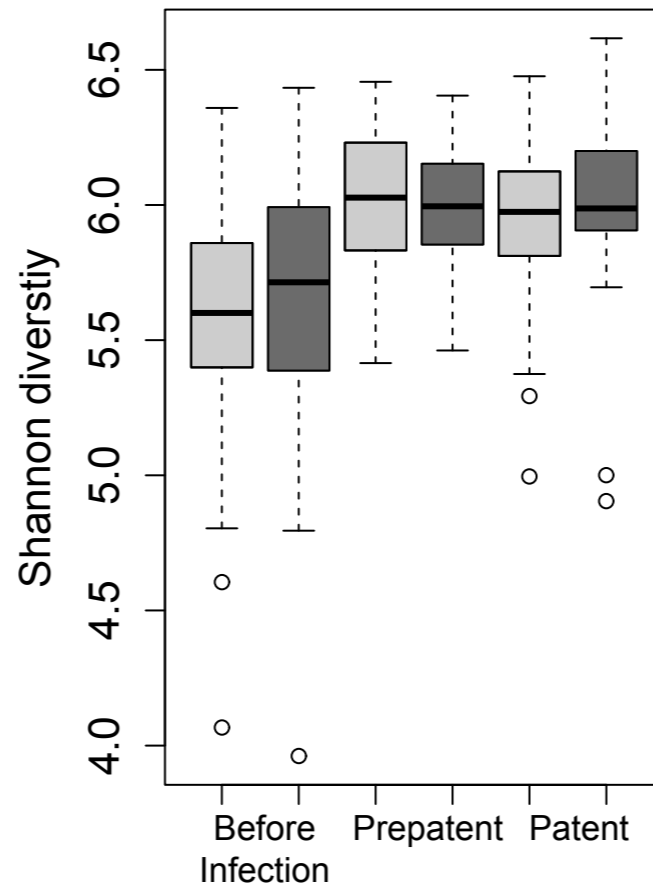

C) *Lactobacillus* abundance

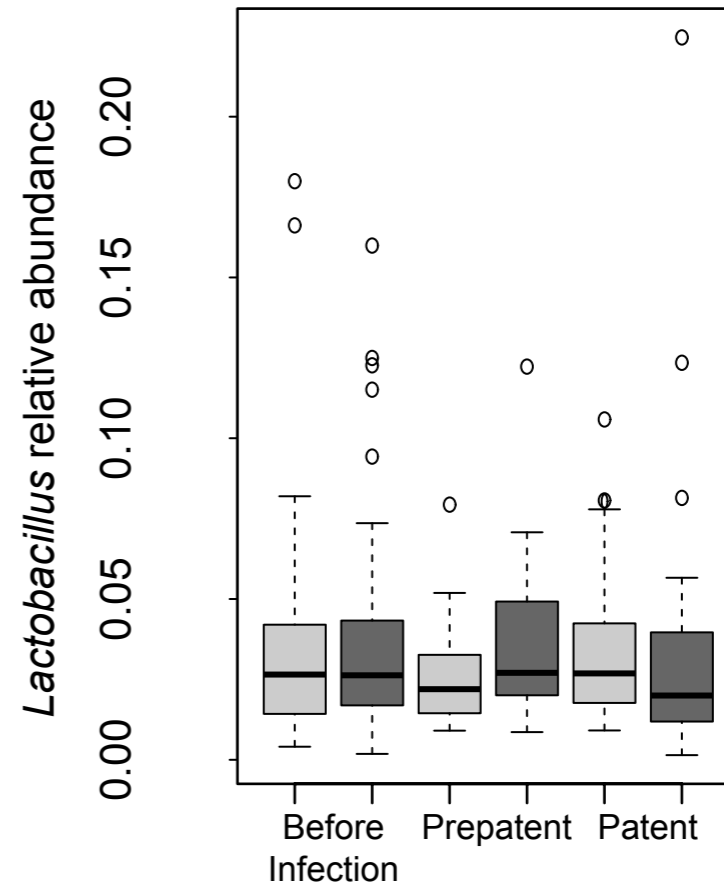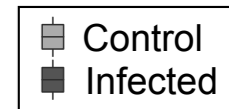

Supplement: S7 Fig — Before infection includes 12 time points, prepatent period includes 4 time points, and patent period includes 11 time points. A and B) Alpha diversity, as measured by the Shannon diversity index, of Bacteroidetes and Firmicutes phyla. A) Shannon diversity within the Bacteroidetes phylum increases in response to helminth infection, but the effect is not significant. B) There is no response in Shannon diversity of the Firmicutes following helminth introduction. C) The relative abundance of the genus Lactobacillus does not change following helminth introduction. (PDF) [file pone.0182205.s007.pdf]
