# Supplementary material for: A benign helminth alters the host immune system and the gut microbiota in a rat model system
Source: PLoS One. 2017 Aug 3;12(8):e0182205. doi: 10.1371/journal.pone.0182205 (PMC5542714; doi:10.1371/journal.pone.0182205)
Supplement: S1 Table — Three-factor PERMANOVA with time period, treatment group, and rat nested within treatment group. Model includes interaction between treatment group and time period. Test used unrestricted permutation of raw data and Type III sum of squares. Bray-Curtis dissimilarity metric. (DOCX) [file pone.0182205.s008.docx]

| Source | df | SS | MS | Pseudo-F | P-value | perms |
| --- | --- | --- | --- | --- | --- | --- |
| Time period | 2 | 4.5957 | 2.2978 | 4.0571 | 0.001 | 998 |
| Treatment group | 1 | 2.0047 | 2.0047 | 2.625 | 0.001 | 908 |
| Rat (Group) | 6 | 4.5824 | 0.76373 | 5.1492 | 0.001 | 998 |
| Time period X Treatment group | 2 | 1.3051 | 0.65257 | 1.1522 | 0.265 | 999 |
| Time period X Rat (Group) | 12 | 6.7981 | 0.56651 | 3.8196 | 0.001 | 997 |
| Residuals | 188 | 27.884 | 0.14832 |  |  |  |
| Total | 211 | 47.897 |  |  |  |  |
