# Supplementary material for: A benign helminth alters the host immune system and the gut microbiota in a rat model system
Source: PLoS One. 2017 Aug 3;12(8):e0182205. doi: 10.1371/journal.pone.0182205 (PMC5542714; doi:10.1371/journal.pone.0182205)
Supplement: S2 Table — Nested PERMANOVA analyses (rat nested within treatment group) were run to differences in community composition similarity before infection (12 time points), in the prepatent period (4 time points), and in the patent period (11 time points). Beta diversity metrics: UW–unweighted UniFrac, W–weighted UniFrac, BC–Bray Curtis). Test used unrestricted permutation of raw data and Type III sum of squares. PERMDISP was used to assess differences in dispersion between treatment groups. (DOCX) [file pone.0182205.s009.docx]

|  |  | PERMANOVA | | | | PERMDISP | |  |
| --- | --- | --- | --- | --- | --- | --- | --- | --- |
| Time period | Metric | between  rats | | between treatment groups | | | between treatment groups | |
|  |  | Pseudo-F | p-value | Pseudo-F | p-value | | p-value | |
| Before infection | BC | 5.01 | 0.001 | 0.98 | 0.462 | | 0.409 | |
| Before infection | UW | 4.44 | 0.001 | 1.62 | 0.075 | | 0.251 | |
| Before infection | W | 5.32 | 0.001 | 0.533 | 0.544 | | 0.348 | |
| Prepatent | BC | 5.23 | 0.001 | 1.97 | 0.075 | | 0.946 | |
| Prepatent | UW | 4.22 | 0.001 | 1.48 | 0.078 | | 0.881 | |
| Prepatent | W | 5.04 | 0.001 | 1.94 | 0.117 | | 0.289 | |
| Patent period | BC | 4.88 | 0.001 | 2.24 | 0.015 | | 0.472 | |
| Patent period | UW | 4.8 | 0.001 | 2.23 | 0.010 | | 0.118 | |
| Patent period | W | 2.72 | 0.001 | 1.58 | 0.176 | | 0.104 | |
